# Antcin C from *Antrodia cinnamomea* Protects Liver Cells Against Free Radical-Induced Oxidative Stress and Apoptosis* In Vitro* and *In Vivo* through Nrf2-Dependent Mechanism

**DOI:** 10.1155/2013/296082

**Published:** 2013-12-11

**Authors:** M. Gokila Vani, K. J. Senthil Kumar, Jiunn-Wang Liao, Shih-Chang Chien, Jeng-Leun Mau, Shen-Shih Chiang, Chin-Chung Lin, Yueh-Hsiung Kuo, Sheng-Yang Wang

**Affiliations:** ^1^Department of Forestry, National Chung Hsing University, Taichung 402, Taiwan; ^2^Department of Cosmeceutics, China Medical University, Taichung 40402, Taiwan; ^3^Graduate Institute of Veterinary Pathology, National Chung Hsing University, Taichung 402, Taiwan; ^4^The Experimental Forest Management Office, National Chung-Hsing University, Taichung 402, Taiwan; ^5^Department of Food Science and Biotechnology, National Chung Hsing University, Taichung 402, Taiwan; ^6^Taiwan Leader Biotech Company, Taipei 24747, Taiwan; ^7^Graduate Institute of Chinese Pharmaceutical Science, China Medical University, Taichung 40402, Taiwan; ^8^Department of Biotechnology, Asia University, Taichung 41354, Taiwan; ^9^Agricultural Biotechnology Research Center, Academia Sinica, Taipei 115, Taiwan

## Abstract

In this study, we investigated the cytoprotective effects of antcin C, a steroid-like compound isolated from Antrodia cinnamaomea against AAPH-induced oxidative stress and apoptosis in human hepatic HepG2 cells. Pretreatment with antcin C significantly protects hepatic cells from AAPH-induced cell death through the inhibition of ROS generation. Furthermore, AAPH-induced lipid peroxidation, ALT/AST secretion and GSH depletion was significantly inhibited by antcin C. The antioxidant potential of antcin C was correlated with induction of antioxidant genes including, HO-1, NQO-1, **γ**-GCLC, and SOD *via* transcriptional activation of Nrf2. The Nrf2 activation by antcin C is mediated by JNK1/2 and PI3K activation, whereas pharmacologic inhibition of JNK1/2 and PI3K abolished antcin C-induced Nrf2 activity. In addition, AAPH-induced apoptosis was significantly inhibited by antcin C through the down-regulation of pro-apoptotic factors including, Bax, cytochrome c, capase 9, -4, -12, -3, and PARP. *In vivo* studies also show that antcin C significantly protected mice liver from AAPH-induced hepatic injury as evidenced by reduction in hepatic enzymes in circulation. Further, immunocytochemistry analyses showed that antcin C significantly increased HO-1 and Nrf2 expression in mice liver tissues. These results strongly suggest that antcin C could protect liver cells from oxidative stress and cell death *via* Nrf2/ARE activation.

## 1. Introduction

Free radical-induced oxidative stress is involved in variety of human pathologies, including brain injury, cardiovascular disease, diabetes, and nephron and hepatic diseases [1]. A basal level of reactive oxygen species (ROS) are generated during normal cellular metabolism; however, cells exposed to toxins or free radical generators produce vast amounts of ROS, which induce lipid peroxidation, protein degradation, and DNA damage [1]. In particular, uncontrolled lipid peroxidation by free radicals is involved in the occurrence of many diseases including arthritis, Alzheimer's, cancer, and cardiovascular and liver diseases [2]. The liver regulates many important metabolic functions, and liver damage can distort these metabolic functions [2]. Despite the fact that acute and chronic liver diseases represent a global concern, modern medical treatments often have limited efficiency [3]. Over the past three to four decades, mounting evidence has shown that dietary phytochemicals are efficacious in preventing oxidative stress-related liver diseases and protecting cells from toxic insult [4–6].

Eukaryotic cells are equipped with a variety of primary and secondary defenses against toxic xenobiotic chemicals or toxicant-induced oxidative stress and their deleterious effects [7]. In addition to this endogenous defense mechanism, induction of phase II enzymes such as hemeoxygenase-1 (HO-1), NAD(P)H: quinone oxidoreductase 1 (NQO1), and glutathione-*S*-transferase (GST) by external factors is an important component of the cellular stress response through which oxidative toxicants can be removed before they damage DNA [8]. Dietary phytochemicals exert protective effects not only by scavenging ROS, but also by inducing de novo expression of antioxidant genes including *γ*-glutamylcysteine synthetase (*γ*-GCLC), HO-1, NQO-1, and GST [9]. Transcriptional activation of antioxidants or detoxifying genes is predominantly regulated by a redox-sensitive transcription factor nuclear factor erythroid 2-related factor-2 (Nrf2). Dietary phytochemicals are able to activate Nrf2 signaling thereby upregulating phase-II enzyme induction in a variety of cells [9].

*Antrodia cinnamomea *is a unique medicinal mushroom used in the traditional Chinese medicine system for the treatment of liver diseases, food and drug intoxication, diarrhea, abdominal pain, hypertension, allergies, skin itching, and tumorigenic diseases [10]. In Taiwan, this mushroom is believed to be one of the most potent liver-protecting herbs. Recent studies have shown that *Antrodia cinnamomea* possesses a number of bioactive properties such as anticancer, antiinflammatory, antioxidant, antihypertensive, antihepatitis B virus replication, hepatoprotective, and neuroprotective functions [10, 11]. *Antrodia cinnamomea* is rich in benzylic compounds, terpenoids, and polysaccharides. Antcin C, a steroid-like compound isolated from the fruiting bodies of *Antrodia cinnamomea *has shown putative cytotoxicity activity on human breast, liver, and leukemia cells [12, 13]. However, the other effects of this potentially beneficial compound have not been investigated. In this study, we demonstrated that antcin C pretreatment protects hepatic cells from free radical-induced oxidative stress and cell death via Nrf2-dependent induction of antioxidant genes *in vitro* and *in vivo*.

## 2. Materials and Methods

### 2.1. Chemicals and Reagents

**Figure 1:**
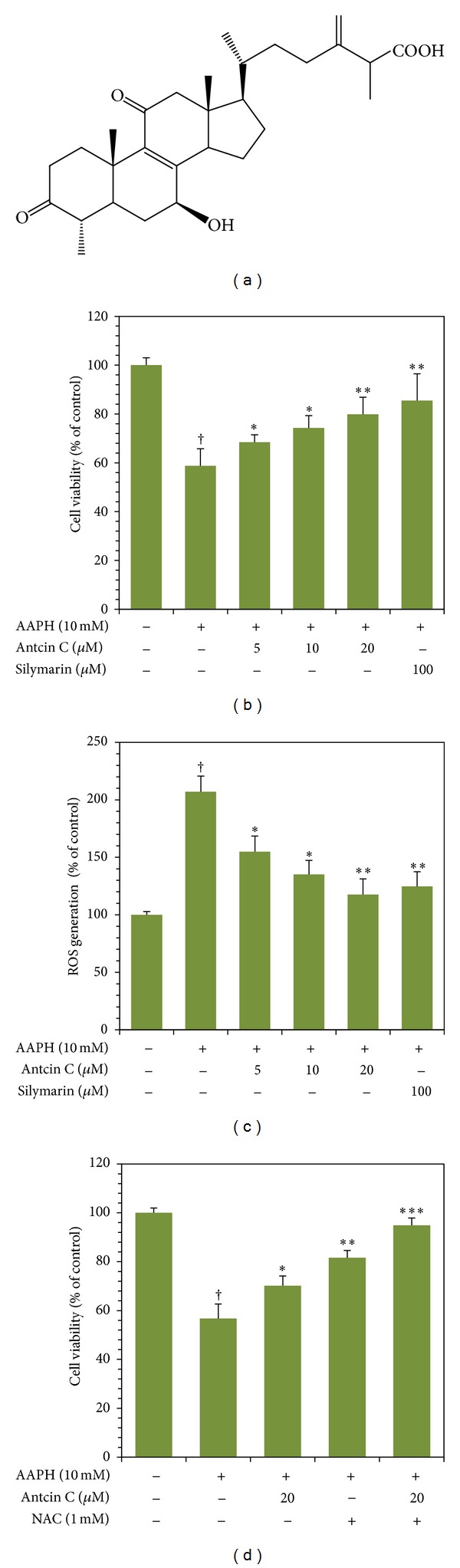
Protective effects of antcin C on AAPH-induced cell death and ROS generation in HepG2 cells. (a) Chemical structure of antcin C. (b) and (d) Cells were pretreated with increasing concentrations of antcin C (5–20 *μ*M), silymarin (100 *μ*M), or NAC (1 mM) for 2 h; then oxidative stress was induced by AAPH (10 mM) for 24 h. Cell viability was measured by MTT assay. Percentage of viable cells was calculated against that of control cells. (c) Cells were pretreated with antcin C (5–20 *μ*M) or silymarin (100 *μ*M) for 2 h, and then ROS generation was induced by AAPH (10 mM) for 30 min. The percentage of fluorescence intensity of the DCF stained cells was quantified using a fluorescence spectrophotometer as described in Materials and Methods. Values represent the mean ± SD of three independent experiments. *P < 0.05, **P < 0.01, and ***P < 0.001 were considered significant for sample versus AAPH. ^†^ *P* < 0.05 was considered significant for control versus AAPH.

Antcin C (Figure 1(a)) was isolated from the fruiting bodies of *Antrodia cinnamomea *as described previously [13]. The purity of antcin C was above 99% according to HPLC and ^1^H-NMR analyses. Dulbecco's modified Eagle's medium (DMEM), fetal bovine serum (FBS), penicillin, and streptomycin were obtained from Gibco/Invitrogen (Carlsbad, CA). Silymarin, 2′,7′-dichlorofluorescein diacetate (DCFH_2_-DA), 2,2′-azobis(2-amidinopropane) dihydrochloride (AAPH), and 3-(4,5-dimethylthiazol-2-yl)-2,5-diphenyltetrazolium bromide (MTT) were purchased from Sigma-Aldrich (St Louis, CA). The list of antibodies used in this study is presented in Table S1 (Supplementary material available online at http://dx.doi.org/10.1155/2013/296082). All the other chemicals used were reagent grade or HPLC grade and were supplied by Merck (Darmstadt, Germany) or Sigma (St. Louis, MO).

### 2.2. Cell Culture and Sample Treatment

Human hepatoma (HepG2) cell line was obtained from American Type Culture Collection (Manassas, VA) and cultured in DMEM supplemented with 10% FBS, glucose, penicillin, and streptomycin. HepG2 cells were grown in 10 cm cell culture dishes and incubated in a humidified atmosphere containing 5% CO_2_ at 37°C. Cultures were allowed to reach 80–90% confluence before experiments were initiated. Cells were seeded at the required density in a culture dish, and incubated in the presence or absence of different concentrations of antcin C (5, 10, and 20 *μ*M) or silymarin (100 *μ*M). Oxidative stress was then induced by exposure to AAPH (10 mM). Control cells received 0.1% DMSO instead of test treatments and/or AAPH. Commercially available silymarin (Sigma) was used as a positive control for all subsequent studies.

### 2.3. Cell Viability Assay

Cell viability was assessed by MTT colorimetric assay. Briefly, 1 × 10^4^ cells/well was seeded in 96-well culture plates. Upon 80% confluence, cells were incubated with increasing concentrations of AAPH (10–100 mM) or antcin C (5–200 *μ*M) for 24 hours. For the additional experiments, cells were incubated with or without the indicated concentrations of antcin C, silymarin, ROS inhibitor, caspase inhibitor, or MAPK inhibitors in the presence or absence of AAPH (10 mM) for 24 hours. Then the culture supernatant was removed and 1 mg/mL of MTT in 100 *μ*L DMEM was added. The MTT formazan crystals were dissolved in 200 *μ*L of DMSO and the samples were measured at 570 nm (A_540_) using an ELISA microplate reader (Bio-Tek Instruments, Winooski, VT). The percentage cell viability (%) was calculated as (A_570_ of treated cells/A_570_ of untreated cells) × 100.

### 2.4. Determination of Intracellular ROS Accumulation

Intracellular ROS accumulation in HepG2 cells was monitored using fluorescent marker DCFH_2_-DA. Briefly, HepG2 cells (1 × 10^5^ cells/well) were seeded in 24-well plates and pretreated with or without increasing concentrations of antcin C (5–20 *μ*M) or silymarin (100 *μ*M) for 2 hours. Oxidative stress was induced by the addition of AAPH (10 mM) into the culture medium for 30 minutes. At the end of the incubation, the culture supernatant was removed and cells were washed twice with PBS. DCFH_2_-DA (10 *μ*M) was mixed with 500 *μ*L DMEM and added to the culture plate. After incubation for 30 minutes, relative fluorescence intensity was quantified using a fluorescence spectrophotometer (Hidex Oy, Turku, Finland) at 485/535 nm (A_485/535_). The percentage of ROS generation (%) was calculated as (A_485/535_ of treated cells/A_485/535_ of untreated cells) × 100.

### 2.5. Determination of Lipid Peroxidation and Intracellular GSH

AAPH-induced lipid peroxidation and GSH depletion was measured in HepG2 cells as described previously [14]. Briefly, HepG2 cells were preincubated with or without various concentrations of antcin C (5–20 *μ*M) or silymarin (100 *μ*M) for 2 hours; then oxidative stress was induced by 10 mM of AAPH for 24 hours. The treated cells were lysed using lysis buffer and centrifuged at 15,000 ×g for 10 min; the obtained supernatant was mixed with an equal amount of tricloroacetic acid (TCA, as a precipitation reagent, provided in the GSH assay kit) and further centrifuged at 15,000 ×g for 5 minutes. The intracellular levels of GSH were measured using nonprotein cell lysates by a commercially available GSH assay kit (Oxis International, Foster City, CA). AAPH-induced lipid peroxidation in HepG2 cells was determined by the formation of malonaldehyde (MDA) in cultured cell lysates using a lipid peroxidation assay kit (Oxford Biomedical Research, Oxford, MI).

### 2.6. Quantification of Hepatic ALT and AST Secretion

Intercellular levels of ALT and AST were measured using commercially available assay kits (Randox Laboratories, Antrim, UK). HepG2 cells at a density of 1 × 10^5^ cell/well were cultured in 12-well plates and preincubated with or without various concentrations of antcin C (5–20 *μ*M) or silymarin (100 *μ*M) for 2 hours; then hepatotoxicity was stimulated by AAPH (10 mM) for 24 hours. The culture supernatant was removed and total ALT and AST were measured at 340 nm using an ELISA microplate reader. Results were expressed as unit per liter (U/L).

### 2.7. Protein Extraction and Western Blot Analysis

HepG2 cells (1 × 10^6^ cells/dish) were cultured in 10 cm dishes and preincubated with or without various concentration of antcin C (5–20 *μ*M) or silymarin (100 *μ*M) for 2 hours, then challenged with AAPH (10 mM) for 30 minutes to 24 hours. Cells were lysed by either mammalian protein extraction reagent or nuclear and cytoplasmic extraction reagent kits (Pierce Biotechnology, Rockford, IL). Protein concentrations were determined using Bio-Rad protein assay reagent (Bio-Rad Laboratories, Hercules, CA). Equal amounts of protein samples (60 *μ*g) were separated by 7–12% SDS-PAGE and the separated proteins were transferred onto polyvinylidene chloride (PVDC) membrane overnight. The transferred protein membranes were blocked with 5% nonfat dried milk for 30 minutes, followed by incubation with specific primary antibodies overnight, and either horseradish peroxidase-conjugated goat anti-rabbit or anti-mouse antibodies for 2 hours. The blots were detected by using VL Chemi-Smart 3000 (Viogene-Biotek, Sunnyvale, CA) with enhanced chemiluminescence (ECL) western blotting reagent (Millipore, Billerica, MA).

### 2.8. Nrf2 Immunofluorescence

HepG2 cells at a density of 2 × 10^4^ cells/well were cultured in DMEM medium with 10% FBS in an 8-well glass Nunc Lab-Tek chamber slide (Thermo Fisher Scientific, Waltham, MA) and pretreated with or without various concentrations of antcin C (5–20 *μ*M), silymarin (100 *μ*M), *N*-acetylcysteine (NAC, 1 mM), or JNK1/2 inhibitor (SP600125, 30 *μ*M) for 2 hours, then exposed to AAPH (10 mM) for 2 hours. After incubation, the culture media was removed and the cells were fixed in 2% paraformaldehyde for 15 minutes, permeabilized with 0.1% Triton X-100 for 10 minutes, washed and blocked with 10% FBS in PBS, and then incubated for 2 hours with anti-Nrf2 primary antibody in 1.5% FBS. The cells were then incubated with the fluorescein isothiocyanate- (FITC-) conjugated secondary antibody for another hour in 6% bovine serum albumin (BSA). Next, the cells were stained with 1 *μ*g/mL DAPI for 5 minutes, washed with PBS, and visualized using a confocal microscope (Leica Microsystems, Buffalo Grove, IL) at 200x magnification.

### 2.9. Nrf2 Knockdown by siRNA

The siRNA transfection was performed using Lipofectamine RNAiMax (Invitrogen) following the manufacturer's instructions. For siRNA transfection, HepG2 cells were grown in DMEM medium containing 10% FBS and plated in 6-well plates to give 40−60% confluence at the time of transfection. The next day, the culture medium was replaced with 500 *μ*L of Opti-MEM (Invitrogen) and cells were transfected using RNAiMAX transfection reagent. For each transfection, 5 *μ*L of RNAiMAX was mixed with 250 *μ*L of Opti-MEM and incubated for 5 min at room temperature. In a separate tube, siRNA (100 pM for a final concentration of 100 nM in 1 mL Opti-MEM) was added to 250 *μ*L of Opti-MEM and the siRNA solution was added to the diluted RNAiMAX reagent. The resulting siRNA/RNAiMAX mixture (500 *μ*L) was incubated for an additional 25 minutes at room temperature to allow complex formation. Subsequently, the solution was added to the cells in the 6-well plates giving a final transfection volume of 1 mL. After incubation for 6 hours, the transfection medium was replaced with 2 mL of standard growth medium and the cells were cultured at 37°C. Cells were replaced with growth medium after 24 hours transfection. After antcin C (20 *μ*M) treatment for 2 hours, cells were induced by AAPH (10 mM) for 2–24 hours and then subjected to western blot and MTT assays.

### 2.10. RNA Extraction and RT- and Q-PCR Analysis

Total RNA extraction and Q-PCR was performed as described previously [14]. In brief, total RNA was extracted from cultured HepG2 cells using the Trizol Reagent. Q-PCR analysis was performed using Applied Biosystems detection instruments and software. Forward and reverse primers (10 *μ*M), and the working solution SYBR green, were used as a PCR master mix, under the following conditions: 96°C for 3 minutes followed by 40 cycles at 96°C for 1 minute, 50°C for 30 seconds, and 72°C for 90 seconds. *β*-Actin, a housekeeping gene, was used as an internal standard to control for variability in amplification because of differences in starting mRNA concentrations. The copy number of each transcript was calculated as the relative copy number normalized by *β*-actin copy number. One microgram of total RNA was subjected to RT-PCR using a BioRad iCycler PCR instrument (Bio-Rad, Hercules, CA) and SuperScript-III One-Step RT-PCR with Platinum Taq DNA Polymerase (Invitrogen); amplification was achieved by 30–38 cycles of 94°C for 45 seconds (denaturing), 60–65°C for 45 seconds (annealing), and 72°C for 1 minute (primer extension). The sequences of the PCR primers were as presented in Table S2.

### 2.11. Animals and Experimental Protocols

AAPH-induced acute hepatotoxicity studies were carried out as per single dose intraperitoneal injection (i.p) as described previously [15, 16] with minor modifications. Four-week-old male ICR mice weighing 25 ± 5 g purchased from Biolasco (Taipei, Taiwan) were used for this study. All animal experiments were conducted in accordance with institutional guidelines for the care and use of laboratory animals and Taiwan laws relating to the protection of animals and were approved by the local ethics committee. Thirty male ICR mice were divided into 6 groups with 5 mice per group. Mice from groups I (control) and II (AAPH alone) received vehicle (2% DMSO in ddH_2_O) throughout the experiment. Groups III–V received 12.5, 50, or 100 mg/kg of antcin C in 100 *μ*L of ddH_2_O, respectively, once a day for 5 days i.p. silymarin was administered to group VI at a dose of 200 mg/kg for 5 days i.p. At the end of the treatments, mice from groups II–VI received 80 mg/kg b.w of AAPH i.p. The single dose and time of injection of AAPH followed a previously described method [16]. After treatment with AAPH for 16 hours, mice were sacrificed by anesthesia with ethyl ether. Blood was collected in EDTA tubes and centrifuged at 15,000 ×g for 10 minutes at 4°C. Serum was obtained and stored at −20°C for further examination. The liver from one mouse in each group was removed and kept at −80°C for further western blot analysis. The remaining livers were washed with PBS and stored in 10% neutral buffered formalin (NBF) for further histopathological analysis.

### 2.12. Determination of Serum ALT, AST, and Cellular MDA and GSH Levels

Serum ALT and AST were measured using commercially available assay kits (Randox Laboratories) as described previously [14]. The intracellular GSH and MDA levels were quantified in nonprotein liver tissue homogenates. Briefly, 1 g of liver tissue was homogenized with 500 *μ*L of PBS and 0.5% Triton X-100 solution. The homogenate were centrifuged in 15,000 ×g for 10 minutes at 4°C. The total content of GSH and MDA in culture supernatant was determined using commercially available EIA kits from Oxis International and Oxford Biomedical Research, respectively.

### 2.13. Immunohistochemical Analysis

Biopsied liver tissues were embedded in paraffin and cut into 3 mm thick sections. To examine the expression of antioxidant markers, the tissue sections were immunohistochemically stained with a mouse monoclonal HO-1 and rabbit polyclonal anti-Nrf-2 antibodies for 2 hours at room temperature. The secondary detection antibody was conjugated to a REAL EnVision Detection System (Dako Cytomation A/S, Denmark).

### 2.14. Statistical Analysis

Data are expressed means ± SD. Statistical analysis of the results were made using Dunnett's test for multiple comparison and Student's *t*-test for single comparison. *P* values of < 0.05, < 0.01, and < 0.001 were considered significant for sample versus AAPH. A *P* value of < 0.05 was considered significant for control versus AAPH.

## 3. Results

### 3.1. Antcin C Protects Hepatic Cells from 2,2′-Azobis(2-amidinopropane) Dihydrochloride-Induced Cell Death *In Vitro*

Prior to the *in vitro* studies, the cytotoxic effect of antcin C was determined using MTT colorimetric assay. Treatment of HepG2 cells with increasing concentrations of antcin C (5–200 *μ*M) for 24 hours did not show any cytotoxic effect up to the concentration of 50 *μ*M. At concentrations greater than 50 *μ*M, cell viability was gradually decreased in a dose-dependent manner (Figure S1(a)). We, therefore, used the noncytotoxic concentrations of antcin C ranging from 5 to 20 *μ*M for our further experiments. We next examined the concentration of AAPH required to induce oxidative stress and cell death in HepG2 cells. HepG2 cells were incubated with increasing concentrations of AAPH (10–100 mM) for 24 h, and cell viability was monitored using MTT assay. As shown in Figure S1(b), cells exposed to AAPH at doses of 10, 20, 50, and 100 mM exhibited a dose-dependent reduction in cell viability to 73.3%, 10%, 3%, and 3%, respectively, compared to control cells (100%). For the protective study, a subcytotoxic concentration of AAPH (10 mM) was administered to generate oxidative stress in HepG2 cells. As shown in Figure 1(b), AAPH-induced reduction in cell viability (65%) was dose dependently inhibited by antcin C. Cell viability of nearly 85% was observed in the antcin C (20 *μ*M) pretreatment group, which is comparable to the known hepatoprotective drug silymarin, which showed 95% cell survival (Figure 1(b)). These results clearly indicate that the exposure of hepatic cells to antcin C confers a significant protective effect against AAPH-induced cell death.

### 3.2. Antcin C Inhibits AAPH-Induced ROS Generation in HepG2 Cells

We hypothesized that AAPH-induced cell death may be due to the induction of intracellular ROS, so next we monitored AAPH-induced intracellular ROS accumulation in HepG2 cells. Results of DCFH_2_-DA fluorescence spectrophotometer analysis showed that exposure of HepG2 cells to AAPH (10 mM) for 30 minutes, significantly increased DCF fluorescence (220%), an indicator of intracellular ROS generation (Figure 1(c)). The increase in intracellular ROS (220%) caused by AAPH was significantly reduced to 168%, 147%, and 131% by 5, 10, and 20 *μ*M of antcin C, respectively (Figure 1(c)). To further confirm our hypothesis that the cytoprotective effect of antcin C is due to the inhibition of ROS generation, HepG2 cells were preincubated with a ROS scavenger NAC with or without antcin C for 2 h, and then the oxidative stress was induced by AAPH for 24 h. Results of MTT assay showed that AAPH-induced reduction in cell numbers (62%) was significantly ameliorated by NAC to 84% (Figure 1(d)). The number of viable cells was further increased to above 95% by cotreatment with NAC and antcin C (Figure 1(d)). These results demonstrate that the cytoprotective effect of antcin C is due to the inhibition of intracellular ROS generation.

### 3.3. Antcin C Inhibits AAPH-Induced Hepatic Transaminase Release, Lipid Peroxidation, and GSH Depletion in HepG2 Cells

Elevated levels of hepatic aminotransferases such as ALT and AST provide a direct evidence of liver injury [17]. Therefore, the hepatic transaminase levels were measured after exposure of HepG2 cells to AAPH. After exposure to AAPH for 24 hours, the amount of ALT was markedly increased to 5.1 U/L from 1.35 U/L. Pretreatment with antcin C significantly inhibited the AAPH-induced ALT elevation. ALT levels were reduced to 3.1 U/L, 2.1 U/L, and 1.5 U/L by treatment with 5, 10, and 20 *μ*M of antcin C, respectively (Table 1). A similar effect was also observed for the release of AST in HepG2 cells. AAPH caused a moderate increase of AST to 4.9 U/L from 3.5 U/L, and the increase in AST level was significantly reduced to 4.5 U/L, 4.1 U/L, and 3.5 U/L by treatment with 5, 10, and 20 *μ*M of antcin C, respectively (Table 1).

Next, we examined the effect of antcin C on AAPH-induced lipid peroxidation and GSH depletion in HepG2 cells. The levels of lipid peroxidation by AAPH were measured by the amount of MDA in culture lysates. An increased amount of intracellular MDA (3.8 mM) was observed after treatment with AAPH. With pretreatment with 20 *μ*M of antcin C, intracellular MDA was reduced to 1.7 mM showing that antcin C significantly inhibited the AAPH-induced lipid peroxidation (Table 1). AAPH treatment also significantly reduced the amount of total GSH in cultured HepG2 cells from 55.8 mM to 25.9 mM, and pretreatment with antcin C significantly inhibited the AAPH-induced GSH depletion to 54, 93, 115 mM by 5, 10, and 20 *μ*M, respectively (Table 1). In addition, we also observed that antcin C treatment not only prevented AAPH-induced GSH depletion, but also dose-dependently increased the intracellular GSH level. When compared to the untreated control, pretreatment with antcin C (20 *μ*M) increased GSH in AAPH-induced HepG2 cells nearly 2-fold (Table 1).

### 3.4. Antcin C Upregulates Antioxidant Gene Expression in HepG2 Cells

**Figure 2:**
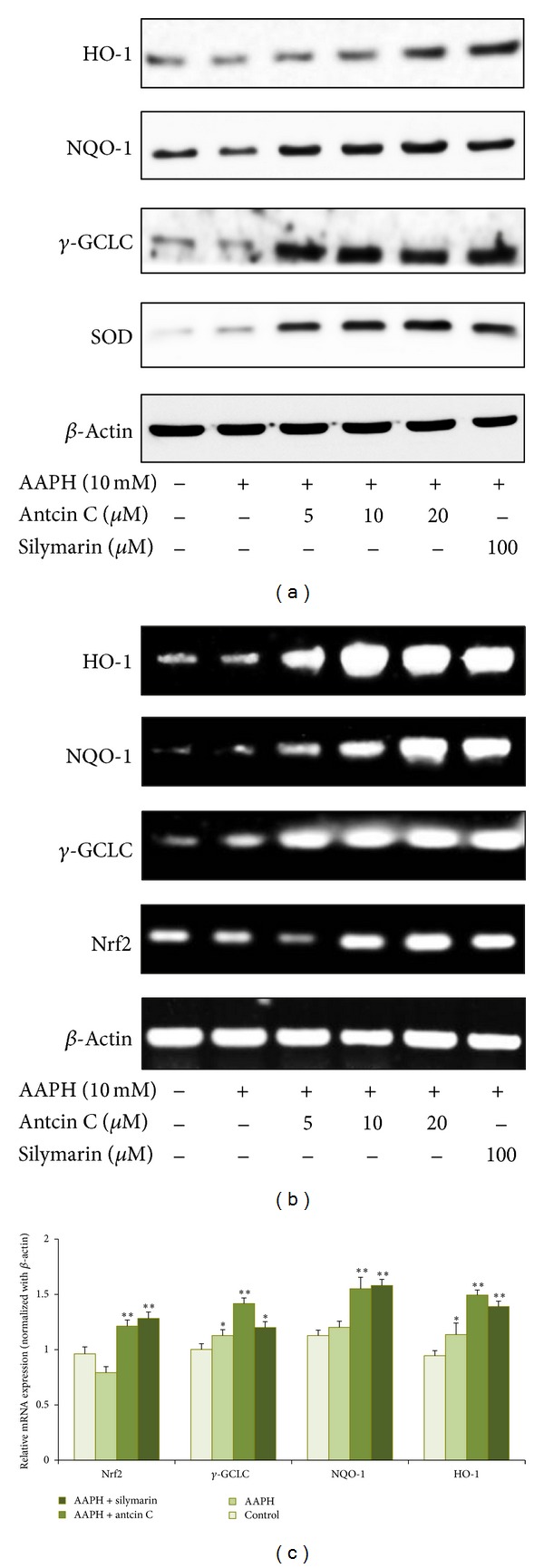
Antcin C induced antioxidant genes expression in AAPH-induced HepG2 cells. (a) HepG2 cells were preincubated with antcin C (5–20 *μ*M) or silymarin (100 *μ*M) for 2 h, and then exposed to AAPH (10 mM) for 24 h. Total cell lysates were prepared and subjected to western blot analysis to monitor the expression levels of antioxidant proteins, including HO-1, NQO-1, *γ*-GCLC, and SOD. *β*-actin served as an internal control. (b) The effect of antcin C on mRNA levels of antioxidant genes. HepG2 cells were pre-incubated with antcin C (5–20 *μ*M) or silymarin (100 *μ*M) for 2 h, and then exposed to AAPH (10 mM) for 6 h. The mRNA levels of HO-1, NQO-1, *γ*-GCLC were semiquantified by RT-PCR analyses. *β*-Actin was used as a loading control. (c) HepG2 cells were pre-incubated with antcin C (20 *μ*M) or silymarin (100 *μ*M) for 2 h, and then exposed to AAPH (10 mM) for 6 h. Q-PCR analysis was performed to monitor the expression levels of HO-1, NQO-1, *γ*-GCLC, and Nrf2 mRNA levels. Values represent the mean ± SD of three independent experiments. *P < 0.05, **P < 0.01, and ***P < 0.001 were considered significant for sample versus AAPH. ^†^ *P* < 0.05 was considered significant for control versus AAPH.

Next, we hypothesized that the inhibitory effect of antcin C on AAPH-induced GSH depletion and/or augmentation of GSH may result from the induction of antioxidant genes such as *γ*-GCLC, HO-1, NQO-1, and SOD, and their corresponding transcription factor Nrf2. As we expected, western blot analysis showed that pretreatment with antcin C significantly increased the protein expression levels of *γ*-GCLC, HO-1, NQO-1, and SOD in a dose-dependent manner (Figure 2(a)). This result was further confirmed by RT-PCR analysis which demonstrated that compared to the untreated control or AAPH treated cells, antcin C-treated cells had significant, dosedependently increased mRNA expression levels of *γ*-GCLC, HO-1, NQO-1, and Nrf2 (Figure 2(b)). In addition, Q-PCR analysis also showed that pretreatment with antcin C significantly increased HO-1, NQO-1, *γ*-GCLC, and Nrf2 mRNA expression in AAPH-induced HepG2 cells (Figure 2(c)).

### 3.5. Antcin C-Induced Upregulation of Antioxidant Genes Is Mediated by the Nrf2 Signaling Pathway

**Figure 3:**
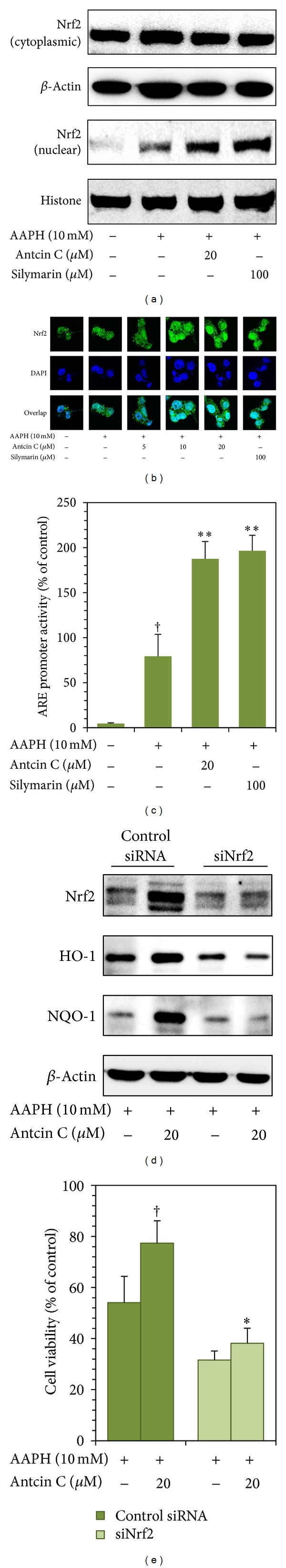
Antcin C promotes transcriptional activation of Nrf2 in AAPH-induced HepG2 cells. (a) Cells were pretreated with antcin C (20 *μ*M) or silymarin (100 *μ*M) for 2 h, and then exposed to AAPH (10 mM) for 1 h. Nuclear and cytoplasmic lysates were prepared and subjected to western blot analysis. The accumulation of Nrf2 in the cytoplasm and the nucleus was monitored. (b) HepG2 cells were pretreated with antcin C (5–20 *μ*M) or silymarin (100 *μ*M) for 2 h, and then exposed to AAPH (10 mM) for 1 h. The protein expression levels of Nrf2 in AAPH-treated HepG2 cells were measured by immunofluorescence using Nrf2 specific primary and fluorescein isothiocyanate-conjugated secondary antibodies (green). The subcellular and nuclear distribution was photographed by fluorescence microscope. DAPI (1 *μ*g/mL) was used to stain the nucleus. (c) HepG2 cells were transiently transfected with ARE plasmids by using lipofectamine and pre-incubated with antcin C (20 *μ*M) or silymarin (100 *μ*M) in the presence or absence of AAPH (10 mM) for 2 h. Cell lysates were mixed with luciferase reagents and quantified using an illuminometer. Relative ARE activity was calculated by dividing the relative luciferase unit (RLU) of treated cells by the RLU of untreated cells. Values represent the mean ± SD of three independent experiments. **P < 0.01 was considered significant for sample versus AAPH. ^†^ *P* < 0.05 was considered significant for control versus AAPH. (d) HepG2 cells were transfected with a specific siRNA against Nrf2 or a nonsilencing control. After 24 h of transfection, the cells were incubated with or without antcin C (20 *μ*M for 2 h) and were induced by AAPH (10 mM) for 2–24 h. Protein expression levels of HO-1, NQO-1, and Nrf2 were monitored by western blot analysis. Cell viability was determined by MTT assay. Values represent the mean ± SD of three independent experiments. *P < 0.05 was considered significant for AAPH versus sample in the control siRNA. ^†^ *P* < 0.05 was considered significant for AAPH versus sample in the siNrf2 cells.

It is well-known that antioxidant genes including *γ*-GCLC, HO-1, NQO-1, and SOD are transcribed by Nrf2, a major transcription factor regulating ARE-driven phase II gene expression. Moreover, the transcriptional activation of Nrf2 is dependent upon the rate of nuclear translocation followed by disassociation from cytoplasmic Keap-1. Therefore, we sought to determine whether antcin C could promote nuclear translocation of Nrf2. HepG2 cells were pre-incubated with antcin C (20 *μ*M) for 2 h, and then exposed to AAPH for 3 h. The protein expression levels of Nrf2 in the cytoplasm and nucleus were examined using specific fractions. As shown in Figure 3(a), compared to untreated control cells, AAPH slightly increased Nrf2 expression in the nucleus, whereas pretreatment with antcin C markedly increased Nrf2 accumulation in the nucleus, which was directly proportional to the decrease in Nrf2 in the cytoplasm. To confirm this effect, immunofluorescence analyses were performed. As shown in Figure 3(b), Nrf2 protein expression was barely observed in control cells, whereas antcin C treatment significantly increased the protein expression of Nrf2 in AAPH-induced HepG2 cells in a dose-dependent manner. In addition, immunofluorescence analysis also demonstrated that antcin C treatment caused a dose-dependent increase of Nrf2 in the nucleus (Figure 3(b)). To demonstrate that antcin C could promote the transcriptional activity of Nrf2 in HepG2 cells, we used the ARE harboring luciferase reporter system. As shown in Figure 3(c), the luciferase activity in HepG2 cells transfected with the ARE reporter construct was significantly increased by AAPH (79 ± 24%) compared to the control (50.7%), whereas a remarkable increase of luciferase activity was observed in the antcin C pretreated cells (187 ± 19%). The difference between the antcin C pretreated cells and the AAPH-challenged cells was statistically significant also indicating that antcin C promotes the transcriptional activation of Nrf2 in HepG2 cells.

### 3.6. Nrf2 Knockdown Diminishes the Protective Effect of Antcin C in HepG2 Cells

To demonstrate the involvement of Nrf2 in antcin C-mediated cytoprotection in hepatic cells, we developed a Nrf2 gene knockdown model in HepG2 cells using siRNA transfection. The knockdown of the Nrf2 gene was confirmed by western blot analysis, which showed that the transfection of siNrf2 (100 pM) completely inhibited the Nrf2 protein expression in HepG2 cells (Figure  2S(a)). As shown in Figure 3(d), significant increases in Nrf2, HO-1, and NQO-1 protein levels were observed in antcin C-treated cells, whereas an antcin C-mediated increase of Nrf2, HO-1, and NQO-1 expression was barely observed in Nrf2 knockdown cells. This result is evidence that the induction of antioxidant genes by antcin C is due to the activation of Nrf2. Transfection of scrambled siRNA (control) did not affect the expression levels of Nrf2, HO-1, or NQO-1 in HepG2 cells (data not shown). To confirm our hypothesis that antcin C protects against hepatic cell death through the up-regulation of Nrf2 and HepG2 cells were transfected with siNrf2, and preincubated with antcin C (20 *μ*M) for 2 hours in the presence of AAPH for 24 hours. Cell viability was measured by MTT assay. Compared to AAPH-treated cells (54%), antcin C pretreatment significantly increased the amount of viable cells to 77% in the control siRNA system (Figure 3(e)); whereas, the percentage of viable cells in Nrf2 knockdown system was further decreased to 32% by AAPH. Antcin C, therefore, failed to protect hepatic cells from AAPH-induced cell death in the Nrf2 knockdown system, as shown by the 38% cell viability observed (Figure 3(e)). Furthermore, the known antioxidant NAC also provided scant protection against AAPH-induced hepatic cell death in Nrf2 knockdown cells (Figure  2S(b)). These data clearly indicate that the cytoprotective effect of antcin C is mediated by Nrf2.

### 3.7. Antcin C-Induced Nrf2 Activation Is Mediated by JNK1/2 and PI3 K Activation in HepG2 Cells

**Figure 4:**
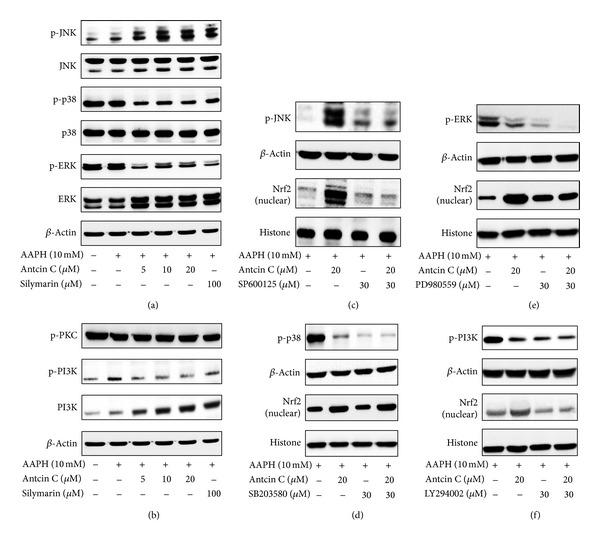
Effects of antcin C on AAPH-induced MAPK activation in HepG2 cells. ((a)–(d)) Cells were pretreated with antcin C (5–20 *μ*M) or silymarin (100 *μ*M) or MAPK inhibitors (30 *μ*M) for 2 h, then exposed to AAPH (10 mM) for 15 min-1 h. Total cell lysates or the nuclear fraction were prepared and subjected to western blot analysis. Phosphorylated and nonphosphorylated forms of p38 MAPK, JNK1/2 and ERK1/2, PKC, and PI3K proteins were examined. *β*-actin was used as a loading control.

It has previously been demonstrated that transcriptional activation of Nrf2 is also dependent on the activation of its upstream kinases including MAPKs, protein kinase C (PKC), and phosphoinositide kinase-3 (PI3 K)/AKT [5]. Therefore, next we examined whether the induction of Nrf2 by antcin C is mediated by the MAPK pathways by measuring phosphorylation of MAPK family proteins, including JNK1/2, ERK1/2, and p38 MAPK in AAPH-induced HepG2 cells. As shown in Figure 4(a), antcin C caused a dose-dependent increase in the phosphorylation of JNK1/2 in HepG2 cells, whereas p38 MAPK and ERK1/2 were significantly inhibited by antcin C. In addition, the total protein levels of JNK1/2, p38 MAPK, and ERK1/2 were unaffected by antcin C (Figure 4(a)). These results suggest that antcin C-induced activation of Nrf2 may be due to the phosphorylation of its upstream kinase JNK1/2. We further investigated the potential effect of antcin C on activation of PKC and PI3 K/AKT in AAPH-induced HepG2 cells. As shown in Figure 4(b), antcin C treatment had no effect on the phosphorylation of PKC, whereas a reduction in phosphorylation of PI3 K was observed. In contrast, the total protein levels of PI3 K were significantly increased by antcin C in a dose-dependent manner. To further confirm this result, prior to AAPH addition, HepG2 cells were pretreated with specific inhibitors for JNK1/2, p38 MAPK, ERK1/2, and PI3 K, (SP600125, SB203580, PD980559, and LY294002, resp.) with or without antcin C for 2 h. Antcin C treatment failed to induce Nrf2 expression in JNK1/2 inhibitor-treated cells (Figure 4(c)); whereas, antcin C-induced Nrf2 expression in HepG2 cells was unaffected by neither p38 MAPK nor ERK1/2 inhibitors (Figures 4(d) and 4(e)). In contrast, antcin C significantly inhibited the phosphorylation of PI3 K in AAPH-induced HepG2 cells. However, pharmacological inhibition of PI3 K significantly decreased antcin C-induced Nrf2 activation in HepG2 cells (Figure 4(f)). These data provide evidence that activation of Nrf2 by antcin C is mediated by JNK1/2 or PI3 K kinase activity.

### 3.8. Antcin C Protects Hepatic Cells from AAPH-Induced Apoptotic Cell Death

**Figure 5:**
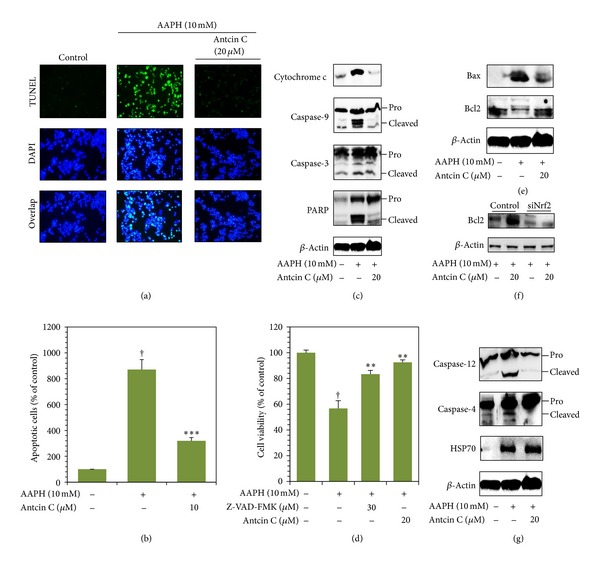
Antcin C protects HepG2 cells from AAPH-induced apoptosis. (a) The DNA fragmentation in AAPH-induced HepG2 cells was determined by TUNEL assay as described in Materials and Methods. The average number of TUNEL-positive cells in microscopic fields (magnification ×200) from three separate samples. (b) Percentages of apoptotic cells were determined by the number of TUNEL positive cells compared to the control. Values represent the mean ± SD of three independent experiments. ***P < 0.001 was considered significant for AAPH versus sample. ^†^ *P* < 0.001 was considered significant for control versus AAPH. ((c), (e), (f)) HepG2 cells were pretreated with antcin C (20 *μ*M) for 2 h, and then exposed to AAPH for 30 min. The effects of antcin C on AAPH-induced cytosolic cytochrome c, caspase 9, caspase 3, PARP, Bcl-2, and Bax (mitochondrial pathway) protein levels were examined by immunoblotting. (g) Caspase 4, caspase 12, and HSP70 (ER stress pathway) was examined using western blot analysis. (d) Cells were pretreated with antcin C with or without caspase-3 inhibitor Z-VAD-FMK (30 *μ*M) for 2 h, and then cell death was induced by AAPH for 24 h. Cell viability was determined by MTT assay. Values represent the mean ± SD of three independent experiments. **P < 0.01 was considered significant for AAPH versus samples. ^†^ *P* < 0.01 was considered significant for control versus AAPH.

Increased intracellular ROS may also trigger apoptotic cell death through the activation of intrinsic apoptotic cascades. DNA fragmentation is known to be a sensitive biological marker of apoptotic cell death. As shown in Figure 5(a), a remarkable increase in TUNEL positive cells was observed in AAPH-treated cells as measured by labeling the 3′-OH ends of the fragmented DNA with dUTP-fluorescein, whereas the increase in TUNEL positive cells was significantly inhibited by antcin C. The percentage of apoptotic cells was quantified by dUTP-fluorescein intensity in control and treated cells. Compared to control cells (100%), the percentage of apoptotic cells was markedly increased to 870% in AAPH-treated cells. However, pretreatment of cells with antcin C (20 *μ*M) significantly reduced the AAPH-induced apoptotic cells to 320% (Figure 5(b)).

### 3.9. Antcin C Inhibits AAPH-Induced Apoptosis through Downregulation of the Intrinsic Apoptotic Cascade in HepG2 Cells

Next, we examined whether AAPH-induced DNA damage and apoptosis in hepatic cells is mediated by the activation of the intrinsic apoptotic pathway through the induction of mitochondrial membrane permeability and release of cytochrome c to the cytosol. Cytochrome c protein level was examined by western blot analysis. When compared to control cells, AAPH treatment caused a significant increase in cytochrome c, and antcin C pretreatment significantly inhibited the AAPH-induced cytochrome c expression in HepG2 cells (Figure 5(c)). We further examined the expression levels of the cytochrome c downstream effector caspases including caspase 9 and caspase 3. Cleavage of caspase 9 and caspase 3 were significantly increased by AAPH treatment. However, antcin C pretreatment significantly prevented the AAPH-induced cleavage of caspase 9 and caspase 3 in HepG2 cells (Figure 5(c)). In addition, following the activation of caspase 3, increase in cleavage of PARP was also observed in AAPH-treated HepG2 cells that was significantly inhibited by antcin C pretreatment (Figure 5(c)). To further confirm that AAPH induces cell death through the activation of caspase signaling events, cells were preincubated with caspase 3 inhibitor Z-VAD-FMK (30 *μ*M) with or without antcin C (20 *μ*M) for 2 hours, then cell death was induced by AAPH for 24 hours. As shown in Figure 5(d), compared with AAPH treated cells (59%), cell viability was increased to 86% in caspase-3 inhibitor treated cells, showing that AAPH-induced cell death was significantly inhibited. The percentage of viable cells was further increased to 94%, when cells were pretreated with combination of antcin C and caspase-3 inhibitor. It is known that the pro-apoptotic protein Bax is activated by ROS and the antiapoptotic protein Bcl2 is suppressed [18]. In this study, we observed that cells exposed to AAPH exhibited significantly increased Bax and decreased Bcl2 expression. Antcin C pretreatment significantly inhibited AAPH-induced upregulation of Bax and downregulation of Bcl2 in HepG2 cells (Figure 5(e)).

In addition, a recent study demonstrated that induction of Nrf2 by antioxidants upregulates Bcl2 expression and maintains cellular homeostasis in hepatic cells [19]. Therefore, we sought to examine whether the upregulation of Bcl2 by antcin C is associated with induction of Nrf2, the Nrf2 knockdown system was performed. As shown in Figure 5(f), the AAPH-induced decrease of Bcl2 expression was unaffected by antcin C in Nrf2 knockdown cells, whereas significant augmentation was observed in control cells. These results strongly suggest (Figure 3(d)) that the cytoprotective effect of antcin C is Nrf2-dependent.

### 3.10. Antcin C-Mediated Inhibition of Apoptosis in AAPH-Induced HepG2 Cells Is Also through the Downregulation of the Endoplasmic Reticulum Stress-Associated Apoptotic Cascade

Prolonged oxidative stress in the endoplasmic reticulum (ER) induced by free radicals results in apoptosis. To determine whether AAPH-induces ER stress in hepatic cells, the activation of ER stress-associated caspases including caspase 4 and caspase 12 was examined. Western blot analysis showed that AAPH treatment appeared to activate caspase 4 and caspase 12 as evidenced by an increase in their corresponding cleavage subunits. Antcin C pretreatment significantly inhibited the AAPH-induced activation of caspase 4 and caspase 12 in HepG2 cells (Figure 5(g)). In addition, we also monitored the effect of antcin C on the protein expression level of ER-resident heat shock protein HSP70 as many eukaryotes react rapidly to ER stress through the induction of heat shock proteins including HSP70 or HSP90 to reestablish normal function. Therefore, the induction of heat shock proteins is a molecular marker of ER-stress. Increased HSP70 expression was observed in antcin C pretreated cells in comparison with the control or AAPH-treated cells (Figure 5(g)). These data further suggest that antcin C pretreatment not only inhibits AAPH-induced apoptosis but also upregulates the expression of ER stress protective gene HSP70. Further to confirm, the involvement of ROS in ER stress and subsequent apoptosis, cells were pretreated with NAC and the activation of caspase 4 was examined. Elimination of ROS by the antioxidant NAC significantly attenuated AAPH-induced activation of ER stress and apoptosis. NAC also markedly prevented the activation of caspase 4 in AAPH-induced HepG2 cells. Essentially, these results suggest that the generation of ROS is an early event that activates and promotes the ER stress-mediated apoptotic pathway in human hepatic HepG2 cells (Figure  S3).

### 3.11. Antcin C Inhibits AAPH-Induced Hepatic Enzyme Release in Mice Serum

Leakage of hepatic enzymes into the bloodstream is one of the hallmarks of liver injury and is considered a first sign of liver damage; therefore, next we examined the levels of hepatic enzymes ALT and AST after AAPH treatment. As shown in Table 2, administration of AAPH (80 mg/kg, b.w) i.p caused severe liver injury in mice, as indicated by increased amounts of ALT (8.5 U/L) and AST (4.7 U/L) in blood serum. However, pretreatment with antcin C for 7 days significantly inhibited AAPH-induced ALT and AST in a dose-dependent manner. Indeed, treatment of antcin C (100 mg/kg, b.w) reduced ALT and AST levels to a basal level: 4.3 U/L and 3.6 U/L, respectively. Moreover, the inhibitory effect of antcin C on AAPH-induced ALT and AST release was comparable to the known hepatoprotective drug silymarin and had levels of 4.6 U/L and 3.7 U/L, respectively at a concentration of 200 mg/kg, b.w (Table 2). In addition, compared to the control group, antcin C pretreatment (100 mg/kg, i.p, once a day for 7 days) caused no change in serum ALT and AST levels (data not shown).

### 3.12. Antcin C Inhibits AAPH-Induced Lipid Peroxidation and GSH Depletion in Mouse Liver Tissue

Next, we observed that AAPH treatment caused rapid consumption of GSH, which led to rapid GSH depletion in mouse liver tissues. The amount of total GSH in the control group was 99.9 mM. After AAPH treatment total GSH rapidly decreased to 64.1 mM (Table 2). Pretreatment with antcin C significantly protected against APPH-induced GSH depletion, as well as dose dependently increasing the amount of total GSH in mice liver tissues. A significant increase in total GSH in mouse blood serum by antcin C was also observed (Table 2). In addition, it is noteworthy that the amount of GSH in the group treated with antcin C (100 mg/Kg) is comparably higher than that in the group treated with silymarin (200 mg/Kg). In order to evaluate the effect of antcin C on AAPH-induced lipid peroxidation in liver tissues, we examined the levels of MDA in mouse liver tissues. As shown in Table 2, compared to control group (7.3 mM), a remarkable increase of MDA (22.1 mM) was observed in the AAPH-treated groups. The AAPH-mediated increase of MDA was significantly inhibited by antcin C in a dose-dependent manner. More precisely, the amount of MDA in the antcin C (100 mg/Kg) pretreated group was similar to that of the control group. In addition, the inhibitory effect of antcin C against AAPH-induced lipid peroxidation was highly comparable to the silymarin-treated group.

### 3.13. Antcin C Upregulates HO-1 and Nrf2 Expression in Mouse Liver Tissues

**Figure 6:**
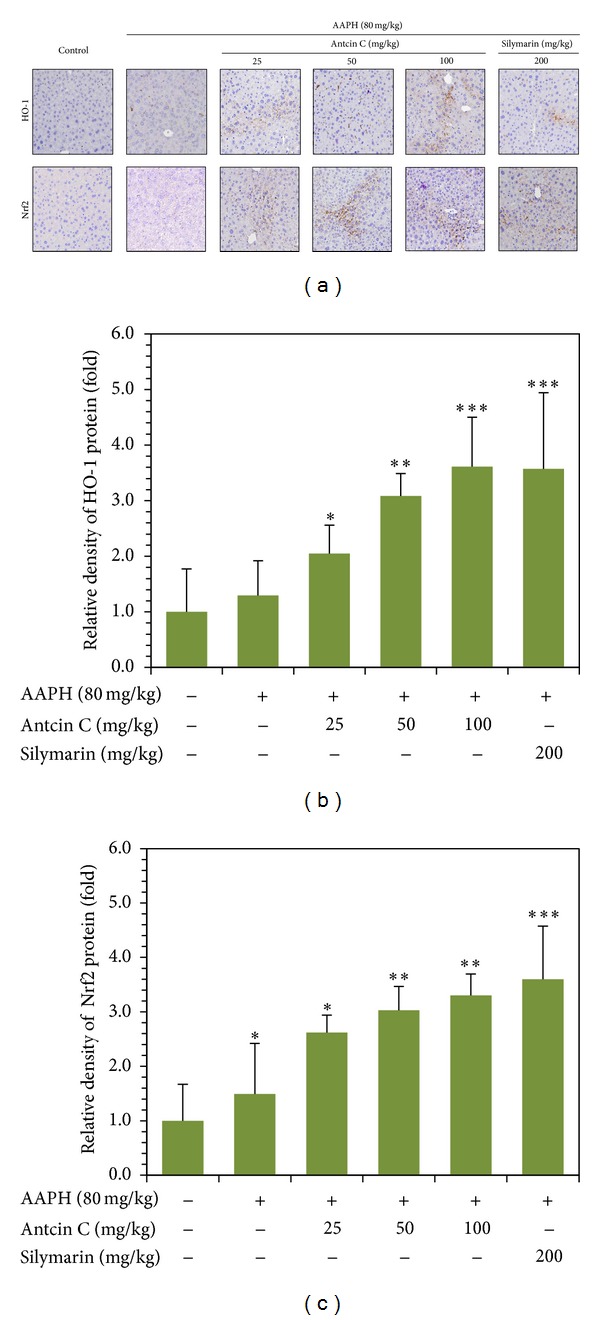
Antcin C upregulates antioxidant genes in AAPH-challenged liver tissues. Mice were pretreated with antcin C (25–200 mg/kg) or silymarin (100 mg/kg) for 5 days, and then challenged with a single dose of AAPH (80 mg/kg). Mice were sacrificed, and the liver sections were subjected to immunohistochemistry to analyse the protein expression levels of HO-1, NQO-1, and Nrf2 using specific antibodies. The relative intensity of antibody positive staining cells was quantified by Image-Pro Plus software. The results are presented as the mean ± SD of three independent experiments. *P < 0.05, **P < 0.01, and ***P < 0.001 were considered significant for sample versus control.

Upregulation of antioxidant genes, including HO-1, NQO-1, and Nrf2, were apparent in antcin C treated cultured HepG2 cells. To delineate this phenomenon *in vivo*, the protein expression levels of HO-1 and Nrf2 were examined by immunohistochemistry and western blot analyses. The results of immunohistochemistry analysis showed that antcin C treatment led to a significant increase in HO-1 and Nrf2 expression compared to the control or AAPH-induced groups (Figure 6(a)). A similar significant increase of HO-1 and Nrf2 expression was also observed in the liver tissues of the silymarin-treated group (Figure 6(a)). More precisely, antcin C treatment induced 2.6-fold, 3.0-fold, and 3.3-fold increases in HO-1 at doses of 25, 50, and 100 mg/kg, respectively (Figure 6(b)). Similarly, 2.0-fold, 3.1-fold, and 3.6-fold increases in Nrf2 protein expression were observed in 25, 50, and 100 mg/kg antcin C pretreatment groups, respectively (Figure 6(c)). These *in vivo *data provide strong evidence that the cytoprotective effect of antcin C results from the upregulation of antioxidant/cytoprotective genes.

## 4. Discussion

Oxidative stress is an abnormal phenomenon occurring inside our body when production of oxygen radicals exceeds their antioxidant capacity. Excess production free radicals and other reactive oxygen species damage essential macromolecules of the cell, leading a number of human diseases including inflammation, cancer, atherosclerosis, rheumatoid arthritis, and neurodegenerative diseases [20]. However, large studies have proved that supplementation with antioxidants particularly dietary phytochemicals reduced the pathogenesis of several human diseases. To study the antioxidative effects of dietary phytochemicals, it is essential to generate free radicals at a controlled and constant rate for specific durations at specific sites [21]. The azo compounds such as 2,2-azobis[2-(2-imidazolin-2-yl)propane] dihydrochloride (AIPH), 2,2-azobis(2-amidinopropane) dihydrochloride (AAPH), and 2,2-azobis[2-methyl-N-(2-hydroxyethyl) propionamide] (AMHP) have been successfully used as free radical initiators in many *in vitro* and *in vivo* studies as the rate and site of free radical formation can be easily controlled using appropriate azo compounds and concentrations [21]. Previous studies have shown that intraperitoneal administration of AAPH to mice caused oxidative damage which could be suppressed by antioxidants [15, 16]. A similar effect was also observed in an *in vitro* system in which AAPH was used to induce oxidative stress in cultured human hepatic (HepG2) cells and pig kidney epithelial (LLC-PK1) cells [22, 23]. Therefore, AAPH-intoxication experiments may be a suitable assay system through which to evaluate the biological activities of dietary phytochemicals and their cytoprotective effects. Here, we employed such an AAPH model system to investigate the cytoprotective effects of antcin C against free radical-induced oxidative stress and cell death. Previous studies reported that AAPH treatment can decrease the viability of hepatic cells [23, 24]. We also observed that HepG2 cells exposed to AAPH for 24 hours had significantly reduced cell viability, consistent with a previous report by Kusumoto et al. [23].

Oxidative stress-induced cell death is associated with increases in reactive oxygen species (ROS), such as hydrogen peroxide, nitric oxide, superoxide anion, hydroxyl radicals, singlet oxygen, and peroxynitrites [25]. It has been reported that AAPH promotes ROS generation can be suppressed by dietary phytochemicals [16]. In this study, we demonstrated that AAPH exposure induced overproduction of ROS in hepatic cells and led to oxidative stress; however, treatment with antcin C significantly suppressed the AAPH-induced ROS generation and increased the survival rate of hepatic cells probably through potent antioxidant activity. Levels of marker enzymes such as AST and ALT in the bloodstream can be used to assess liver function, and their presence in the serum may provide evidence of organ dysfunction [26]. In addition, the levels of AST and ALT in the media of cultured cells are considered to be similar to those in the bloodstream. Previous studies have shown that AAPH exposure caused liver injury and dysfunction, as indicated by elevated levels of serum enzymes through cellular leakage and loss of functional integrity of the hepatic membrane [27, 28]. In this study, increased amounts of ALT and AST were observed in AAPH-induced cell culture media. However, pretreatment with antcin C significantly protected AAPH-induced AST and ALT elevation in the culture media. *In vivo* study also showed that pretreatment with antcin C effectively protected mice against AAPH-induced liver damage by reducing elevated serum AST and ALT.

Exposure to chemical or physical agents triggers membrane free radical reactions in living cells, which accelerates lipid peroxidation. Lipid peroxidation of polyunsaturated fatty acid produces ROS and toxic aldehydes such as 4-HNE and MDA [29]. Therefore, the concentration of MDA in cells or tissue lysates is considered to be a major lipid peroxidation maker. Park et al. [30] demonstrated that U937 cells exposed to AAPH show oxidative stress and DNA damage followed by ROS generation and lipid peroxidation. In the present investigation AAPH induced significant lipid peroxidation in cultured hepatic cells as well as mouse liver tissues as evidenced by increased amounts of MDA. Antcin C pretreatment significantly decreased MDA production in AAPH-treated hepatic cells and mouse liver, likely due to the powerful antioxidant and free radical scavenging activity of antcin C. GSH can act as a nonenzymatic antioxidant by direct interaction of its sulfhydryl group with ROS or it can be involved in the enzymatic detoxification of ROS, as a cofactor or coenzyme [31]. Therefore, GSH is also considered to be an oxidative stress marker. In this study we found that AAPH exposure decreased GSH levels in the cultured human hepatic cells and mice liver tissues, which is in agreement with a previous report [23]. Interestingly, we found that antcin C markedly increased levels of GSH in AAPH-treated human hepatic cells and mouse liver tissues.

Besides exogenous antioxidant defense, the body depends on several endogenous defense mechanisms to protect against free radical-induced cell damage. Among these antioxidant molecules, the phase II enzymes including HO-1, NQO-1, SOD, GST, and *γ*-GCLC play important roles in the exclusion of reactive oxygen species [32]. In this study, the effects of antcin C can be explained not only on the basis of nonenzymatic actions but also on the basis of its effect on enzymatic actions involved in the Nrf2-dependent signaling pathway. We found that antcin C treatment significantly upregulated antioxidant genes such as HO-1, NQO-1, *γ*-GCLC, and SOD at both the transcriptional and translational levels. Nrf2, a basic leucine zipper (bZIP) transcription factor that belongs to the CNC family, plays a central role in transcriptional regulation of phase II enzymes [33]. Upon stimulation, Nrf2 disassociates from its cytosolic inhibitor Keap-1, translocates into the nucleus, and binds to the *cis*-acting ARE in the promoter regions of many phase II enzymes, including HO-1 [5]. ARE domains seem to play a major role in the induction of antioxidant gene promoters by dietary phytochemicals as evidenced by the finding that deletion of ARE-site containing E1 and E2 regions blunts induction [34]. In this study, we demonstrated that antcin C induces both the nuclear translocation of Nrf2 and the subsequent ARE-dependent transcriptional activation in human hepatic HepG2 cells.

In addition, phosphorylation of Nrf2 at serine and threonine residues by kinases such as PI3 K, PKC, and various MAPKs including JNK1/2, ERK1/2, and p38 MAPK is assumed to facilitate the dissociation of Nrf2 from Keap-1 and subsequent translocation to the nucleus [5, 9]. In this study, we found that phosphorylation of JNK1/2 was significantly increased in antcin C-treated cells compared to control or AAPH-only treated cells, whereas phosphorylation of PI3 K, p38 MAPK, and ERK1/2 was inhibited by antcin C. Notably, antcin C caused a substantial increase in total PI3 K expression in HepG2 cells. On the other hand, treatment of HepG2 cells with SP600125, a pharmacological inhibitor of JNK1/2 prevented antcin C-mediated upregulation of Nrf2 in HepG2 cells. The pharmacological inhibition of PI3 K by LY294002 also inhibits the antcin C-mediated upregulation of Nrf2, but antcin C itself decreases phosphorylation of PI3 K, which suggests that phosphorylation of PI3 K is not involved in Nrf2 activation, whereas increase in total PI3 K may be involved. Previous studies also support the notion that exposure of HepG2 cells to dietary phytochemicals induces transcriptional activation of Nrf2 accompanied by activation of JNK1/2 and PI3 K [35, 36].

ROS generation is normally counter balanced by the action of antioxidant enzymes and other redox molecules. However, generation of excess amounts of ROS reduces mitochondrial membrane potential and is implicated in a variety of apoptotic cascades [25]. The balance between the pro-apoptotic proteins Bax/Bad and the antiapoptotic proteins Bcl2/Bcl-xL determines whether cells undergo apoptosis or abort the process [37]. Previous studies have shown that AAPH induces the intrinsic apoptotic pathways followed by the induction of intracellular ROS, mitochondrial permeability transition, and release of cytochrome c [23, 38]. DNA fragmentation is one of the most often used techniques in the study of cell death. Internucleosomal DNA fragmentation can be visualized by TUNEL end labeling and is considered a biochemical hallmark of apoptosis [39]. In this study, TUNEL assay showed that AAPH exposure caused DNA fragmentation as evidenced by increasing amounts of TUNEL positive cells. Pretreatment with antcin C significantly inhibited AAPH-induced DNA fragmentation and apoptotic cell death. AAPH upregulated the expression of Bax in addition to downregulating Bcl2 in HepG2 cells, and enhancing cytochrome c, caspase 9, caspase 3, and PARP activation. Antcin C, however, could successfully inhibit all these mitochondrial-dependent events in AAPH-induced hepatic cells, suggesting its protective effect. A recent study by Niture and Jaiswal [19] demonstrated that protein expression of Bcl2 is upregulated by Nrf2 in human hepatic cells. Our findings also correlated with this notion that siRNA-mediated downregulation of Nrf2 let to decreased antcin C-induced Bcl2 expression in HepG2 cells. These data provide positive evidence of the role of Nrf2 in control of Bcl-2 expression and apoptosis in AAPH-induced human hepatic HepG2 cells.

In addition to mitochondria-dependent apoptosis, recent studies have demonstrated that ER stress induces apoptosis without involvement of the mitochondria [40]. ER stress can be triggered by activation of caspase 12 and caspase 4 [40, 41]. In this study, we first established that AAPH could cause apoptosis in liver cells through the induction of ER stress as evidenced by increased activation of caspase 4 and caspase 12. However, antcin C treatment significantly inhibited the AAPH-induced ER stress and apoptosis through the inhibition of caspase 4 and caspase 12 activation followed by suppression of ROS generation. This result was further confirmed by removal of ROS by the antioxidant NAC which significantly attenuated AAPH-induced activation of caspase 4 and caspase 12. These results strongly suggest that the generation of ROS is an early event that initiates and activates the ER stress-mediated apoptotic pathway in human hepatic HepG2 cells.

**Figure 7:**
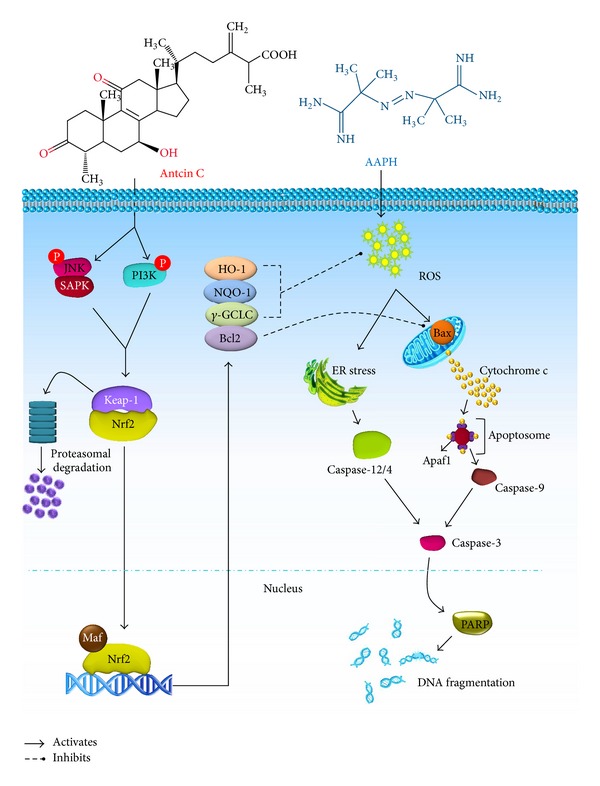
Schematic representation of antcin C-induced upregulation of antioxidative gene expression via the Nrf2/ARE signaling pathway and suppression of AAPH-induced apoptosis in human hepatic HepG2 cells.

In conclusion, our results demonstrate that antcin C induces a Nrf2-mediated group of antioxidant enzymes *via *increase of JNK/1/2 and PI3 K/AKT activities. Also, this study revealed that antcin C protects human hepatic HepG2 cells against oxidative stress-induced cell death through dysregulation of apoptosis-related genes via elevated activation of Nrf2, which appears to be responsible for the induction of Bcl2 expression. Our results also indicate an important role for ROS and ER stress in AAPH-induced apoptosis and suggest that inhibition of cell death by antcin C is also associated with elimination of oxidative stress in the ER followed by inhibition of intracellular ROS (Figure 7). These *in vitro* and* in vivo* results thus expand our understanding of the role of antcin C in liver protection and potentially assist in the identification of new therapeutic strategies for diseases caused by chemical toxicants and other environmental stress.

## 5. Highlights

1. Antcin C protects hepatic cells from oxidative stress through Nrf2/ARE activity.
2. Antcin C suppresses free radical-induced apoptosis via suppressing ROS generation.
3. The activation of Nrf2 by antcin C is mediated by JNK/PI3K signaling cascades.
4. Antcin C prevents cell death through the up- and downregulation of Bcl-2 and Bax.

## Supplementary Material

Additional information on this article including, list of antibodies and oligonucleotides were summarized in Table S1 and Table S2, respectively. Figure S1 (a and b) shows effect of cell viability on AAPH and antcin C. Figure S2 shows (a) Nrf2 knock-down by siNrf2 and (b) treatment with NAC or antcin C failed to prevent AAPH-induced cell death in Nrf2 knock-down cells. Figure S3 shows NAC treatment prevents AAPH-induced caspase-4 activation as well as cell death in hepatic cells. These data can be found in online appendix at http://dx.doi.org/10.1155/2013/296082

## Figures and Tables

**Table 1:** Effect of antcin C on AAPH-induced hepatic enzyme leakage, lipid peroxidation, and glutathione depletion in human hepatic HepG2 cells.

Parameters		AAPH (10 mM)				
			Antcin C (*μ*M)			Silymarin (*μ*M)
	Control	2.5	5	10	100
ALT (U/L)	1.3 ± 0.5	3.2 ± 1.9^†^	2.5 ± 0.6*	2.0 ± 0.1**	1.4 ± 0.1***	0.1 ± 0.2***
AST (U/L)	3.4 ± 0.1	4.8 ± 0.1^†^	4.5 ± 0.5*	4.1 ± 0.5**	3.5 ± 0.1***	2.4 ± 0.2***
GSH (mM)	50.5 ± 5.3	21.7 ± 4.2^†^	50.4 ± 4.5*	88.4 ± 4.8**	111.5 ± 3.7***	123.7 ± 1.0***
MDA (mM)	1.2 ± 0.2	3.5 ± 0.3^†^	2.7 ± 0.2*	2.1 ± 0.3**	1.5 ± 0.2***	1.4 ± 0.2***

Values represent the mean ± SD of three independent experiments. *P < 0.05, **P < 0.01, ***P < 0.001 was considered significant for sample versus. AAPH. ^†^ *P* < 0.05 was considered significant for control versus AAPH.

**Table 2:** Effect of antcin C on hepatic enzyme leakage, lipid peroxidation and glutathione depletion in AAPH-challenged mouse serum or liver tissues.

Parameters			AAPH (80 mg/Kg b.w)			
			Antcin (mg/Kg b.w)			Silymarin (mg/Kg b.w)
	Control		25	50	100	200
Serum ALT (U/L)	4.1 ± 0.2	6.2 ± 2.3^†^	4.9 ± 1.4**	4.6 ± 0.1***	4.2 ± 0.1***	4.4 ± 0.2***
Serum AST (U/L)	3.7 ± 0.2	4.2 ± 0.5^†^	4.1 ± 0.7*	3.7 ± 0.2***	3.4 ± 0.2***	3.6 ± 0.1***
Hepatic GSH (mM)	97.2 ± 2.7	54.3 ± 9.9^†^	90.9 ± 7.9**	125.6 ± 8.4***	162.3 ± 18.8***	216.5 ± 38.2***
Serum GSH (mM)	7.5 ± 2.6	7.9 ± 7.3	21.1 ± 7.2**	49.9 ± 8.0***	92.6 ± 2.1***	81.9 ± 1.5***
Hepatic MDA (mM)	6.2 ± 1.1	20.9 ± 1.2^†^	13.3 ± 1.1*	10.7 ± 1.2**	6.4 ± 1.2***	6.6 ± 1.3***

Values represent the mean ± SD of three independent experiments. *P < 0.05, **P < 0.01, and ***P < 0.001 were considered significant for sample versus. AAPH. ^†^ *P* < 0.05 was considered significant for control versus AAPH.

## Abbreviations

AAPH: 2,2′-azobis(2-amidinopropane) dihydrochloride
ALT: Alanine aminotransferase
ARE: Antioxidant responsive element
AST: Aspartate aminotransferase
ATCC: American type culture collection
BSA: bovine serum albumin
DAPI: 4′,6-Diamidino-2-phenylindole
DCFH_2_-DA: 2′,7′-Dichlorofluorescein diacetate
DMEM: Dulbecco's modified Eagle's medium
DMSO: Dimethyl sulfoxide
ECL: Enhanced chemiluminescence
ELISA: Enzyme-linked immunosorbent assay
ERK: Extracellular signal regulated kinase
FBS: Fetal bovine serum;
GSH: Glutathione
^1^H-NMR: Proton nuclear magnetic resonance
HO-1: Hemeoxygenase-1
HPLC: High performance liquid chromatography
ICR: Institute of Cancer Research
JNK: c-Jun N-terminal kinase
MAPK: Mitogen activated protein kinase
MDA: Malondialdehyde
MTT: 3-(4,5-dimethylthiazol-2-yl)-2,5-diphenyltetrazolium bromide
NAC: *N*-acetylcysteine
NE-PER: Nuclear extract-protein extraction reagent
Nrf-2: Nuclear factor erythroid 2-related factor 2
NQO-1, NAD(P)H: Quinone oxidoreductase-1
PARP: Poly(ADP ribose) polymerase
PBS: Phosphate buffer saline
PVDF: Polyvinylidene difluoride
q-PCR: Quantitative-polymerase chain reaction
ROS: Reactive oxygen species
RT-PCR: Reverse transcriptase-polymerase chain reaction
S.D.: Standard deviation
SDS-PAGE: Sodium dodecyl sulphate-polyacrylamide gel electrophoresis
*γ*-GCLC: *γ*-Glutamylcysteine synthetase.
