# Supplementary material for: Antcin C from Antrodia cinnamomea Protects Liver Cells Against Free Radical-Induced Oxidative Stress and Apoptosis In Vitro and In Vivo through Nrf2-Dependent Mechanism
Source: Evid Based Complement Alternat Med. 2013 Dec 11;2013:296082. doi: 10.1155/2013/296082 (PMC3874316; doi:10.1155/2013/296082)
Supplement: Supplementary file 1 — Additional information on this article including, list of antibodies and oligonucleotides were summarized in Table S1 and Table S2, respectively. Figure S1 (a and b) shows effect of cell viability on AAPH and antcin C. Figure S2 shows (a) Nrf2 knock-down by siNrf2 and (b) treatment with NAC or antcin C failed to prevent AAPH-induced cell death in Nrf2 knock-down cells. Figure S3 shows NAC treatment prevents AAPH-induced caspase-4 activation as well as cell death in hepatic cells. These data can be found in online appendix at http://dx.doi.org/10.1155/2013/296082 [file 296082.f1.pdf]

Supporting Information

Antcin C from *Antrodia cinnamomea* Protects Liver Cells Against Free Radical-Induced Oxidative Stress and Apoptosis *In Vitro* and *In Vivo* through Nrf2-Dependent Mechanism

M. Gokila Vani[1], K.J. Senthil Kumar[2], Jiunn-Wang Liao[3], Shih-Chang Chien[4], Jeng-Leun Mau[5], Shen-Shih Chiang[5], Yueh-Hsiung Kuo[6], Sheng-Yang Wang[1,7]*

[1]*Department of Forestry, National Chung Hsing University, Taichung, Taiwan.*

[2]*Department of Cosmeceutics, China Medical University, Taichung, Taiwan.*

[3]*Graduate Institute of Veterinary Pathology, National Chung Hsing University, Taichung, Taiwan.*

[4]*The Experimental Forest Management Office, National Chung-Hsing University*

[5]*Department of Food Science and Biotechnology, National Chung Hsing University, Taichung, Taiwan.*

[6]*Graduate Institute of Chinese Pharmaceutical Science, China Medical University, Taichung, Taiwan.*

[7]*Agricultural Biotechnology Research Center, Academia Sinica, Taipei, Taiwan.*

*Corresponding author. Tel.: +886-4-22840345-138., Fax.: +886-4-22873628

E-mail address: taiwanfir@dragon.nchu.edu.tw (S-Y Wang).

Table S1 Antibodies used for immunoblotting

Name of antibody	Supplier
Rabbit polyclonal anti-Nrf-2	Abcam (Cambridge, UK)
Mouse monoclonal anti-HO-1	Abcam (Cambridge, UK)
Rabbit polyclonal anti-caspase-4	BioVision (Milpitas, CA)
Human monoclonal anti-HSP70	BD Biosciences (San Jose, CA)
Rabbit monoclonal Histone H3	Cell Signaling Technology (Danvers, MA)
Rabbit monoclonal anti-JNK	Cell Signaling Technology (Danvers, MA)
Rabbit monoclonal anti-p-JNK	Cell Signaling Technology (Danvers, MA)
Rabbit monoclonal anti-p38MAPK	Cell Signaling Technology (Danvers, MA)
Rabbit monoclonal anti-p-p38MAPK	Cell Signaling Technology (Danvers, MA)
Rabbit monoclonal anti-p-ERK1/2	Cell Signaling Technology (Danvers, MA)
Rabbit monoclonal anti ERK1/2	Cell Signaling Technology (Danvers, MA)
Rabbit polyclonal anti-p-PI3K	Cell Signaling Technology (Danvers, MA)
Rabbit monoclonal anti-PI3K	Cell Signaling Technology (Danvers, MA)
Mouse monoclonal anti-caspase-9	Cell Signaling Technology (Danvers, MA)
Rabbit monoclonal anti-PARP	Cell Signaling Technology (Danvers, MA)
Anti-rabbit IgG- HRP conjucated	Cell Signaling Technology (Danvers, MA)
Anti-mouse IgG- HRP conjucated	Cell Signaling Technology (Danvers, MA)
Rabbit polyclonal anti-γ-GCLC	GeneTex (Irvine, CA)
Rabbit polyclonal anti-Cu/Zn-SOD	Millipore (Billerica, MA)
Rabbit polyclonal anti-caspase-12	Millipore (Billerica, MA)
Goat polyclonal anti-NQO-1	Santa Cruz Biotechnology (Heidelberg, Germany)
Mouse monoclonal anti-β-actin	Santa Cruz Biotechnology (Heidelberg, Germany)
Mouse monoclonal anti-p-PKC	Santa Cruz Biotechnology (Heidelberg, Germany)
Rabbit polyclonal anti-cytochrome c	Santa Cruz Biotechnology (Heidelberg, Germany)
Rabbit polyclonal anti-caspase-3	Santa Cruz Biotechnology (Heidelberg, Germany)
Mouse monoclonal anti-Bax	Santa Cruz Biotechnology (Heidelberg, Germany)

Anti-goat IgG-HRP conjucated	Santa Cruz Biotechnology (Heidelberg, Germany)

Table S2 Oligonucleotides used for RT-PCR and Q-PCR

Name	Sequence	Reference
HO-1	F: 5'- TGC GGT GCA GCT CTT CTG-3'	(Liu et a., 2004)
	R: 5'- GCA ACC CGA CAG CAT GC-3'	
NQO-1	F: 5'- CGC AGA CCT TGT GAT ATT CCA G-3'	(Abdelhamid et al., 2010)
	R: 5'- CGT TTC TTC CAT CCT TCC AGG-3'	
γ-GCLC	F: 5'- AGT TCA ATA CAG TTG AGG-3'	(Kim et al., 2006)
	R: 5'- TAC TGA TCC TAT AGT TAT-3-3'	
Nrf2	F: 5'- AAC CAC CCT GAA AGC ACA GC-3'	(Abdelhamid et al., 2010)
	R: 5'- TGA AAT GCC GGA GTC AGA ATC-3'	
β-actin	F: 5'- ACC CAC ACT GTG CCC ATC TA-3'	(Liu al., 2004)
	R: 5'- CGG AAC CGC TCA TTG CC-3'	

References

Liu, L.; Yan, H.; Zhang, W.; Yao, P.; Zhang, X.; Sun, X. Induction of heme oxygenase-1 inhuman hepatoma cells to protect them from ethanol-induced cytotoxicity. *Biomed. Environ. Sci***17:** 315-326; 2004.

Abdelhamid, G.; Anwar-Mohamed, A.; Elmazar, M. M.; El-Kadi, A. O. Modulation of NAD(P)H:quinone oxidoreductase by vanadium in human hepatoma HepG2 cells. *Toxicol. In Vitro***24:**1554-61; 2010.

Kim, J. Y.; Yim, J. H.; Cho, J. H.; Kim, J. H.; Ko, J. H.; Kim, S. M.; Park, S.; Park, J. H. Adrenomedullin regulates cellular glutathione content via modulation of gamma-glutamate-cysteine ligase catalytic subunit expression. Endocrinology **147:**1357-64; 2006.

FIGURE 1S

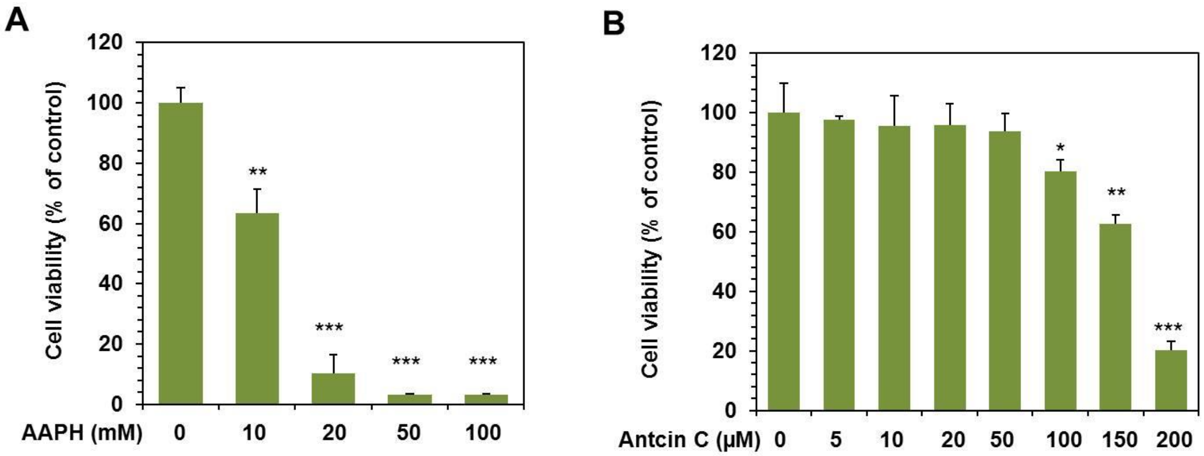

FIGURE S1: Cytotoxic effect of antcin C and AAPH on cultured human hepatoma HepG2 cells. Cell viability was measured by MTT assay as described in Materials and Methods. Cells were

treated with increasing concentrations of AAPH and antcin C for 24 h. (**A**) Cells treated with various concentrations of AAPH for 24 h. (**B**) Cells were treated with various concentrations of antcin C for 24 h. Percentage of viable cells was calculated with control cells. Values represent the mean ± S.D of three experiments. *P < 0.05, **P < 0.01 and ***P < 0.001 was considered significant for control cells.

FIGURE S2.

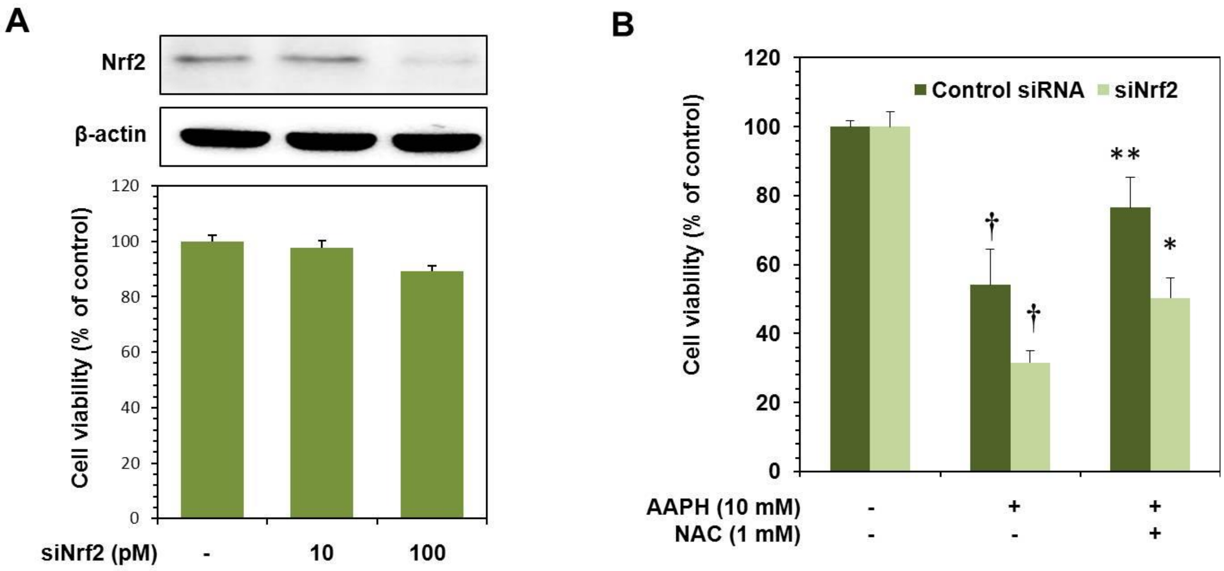

FIGURE S2: SiNrf2-mediated knockdown of Nrf2 in human hepatic HepG2 cells. (A) Cells were transfected with increasing concentrations of siNrf2 for 24 h. The protein level of Nrf2 was examined by western blotting and cell survival was measured by MTT assay. (B) HepG2 cells were transfected with specific siRNA against Nrf2 or a non-silencing control. Following transfection for 24 h, the cells were incubated with or without NAC (1 mM) and AAPH (10 mM) for 24 h. Cell survival was examined by MTT assay. Values represent the mean ± SD of three independent experiments. $^*P < 0.05$, $^{**}P < 0.01$ was considered significant for AAPH *vs.* samples. $^{\dagger}P < 0.01$ was considered significant for control *vs.* AAPH.

FIGURE S3.

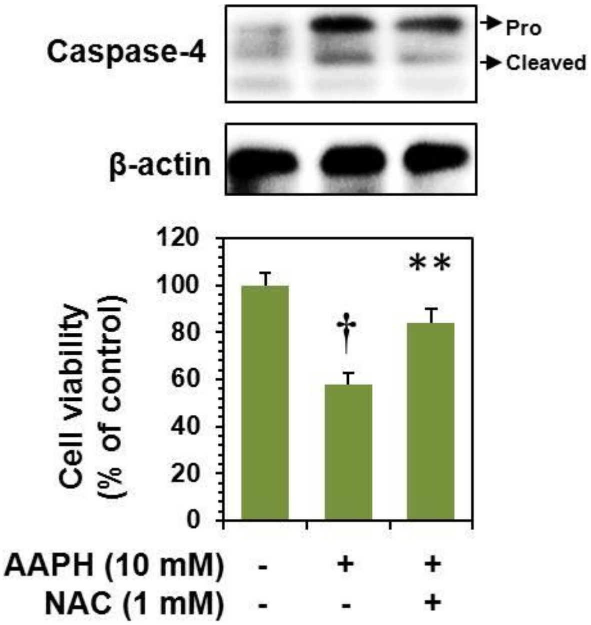

FIGURE S3: NAC protects HepG2 cells from AAPH-induced apoptosis through suppression of ER stress-mediated apoptosis followed by inhibition of ROS. Cells were pretreated with NAC for 2 h, and then oxidative stress was induced by AAPH for 2-24 h. The effects of NAC on AAPH-induced ER-stress marker protein caspase 4 were examined by western blotting and cell survival was examined by MTT assay. Values represent the mean ± SD of three independent experiments. ** $P < 0.01$ was considered significant for AAPH *vs.* samples. $^{\dagger}P < 0.05$ was considered significant for control *vs.* AAPH.
