# Supplementary material for: Antcin C from Antrodia cinnamomea Protects Liver Cells Against Free Radical-Induced Oxidative Stress and Apoptosis In Vitro and In Vivo through Nrf2-Dependent Mechanism
Source: Evid Based Complement Alternat Med. 2013 Dec 11;2013:296082. doi: 10.1155/2013/296082 (PMC3874316; doi:10.1155/2013/296082)
Supplement: Supplementary file 2 [file 296082.f2.pdf]

Supporting Information

Antcin C from *Antrodia cinnamomea* protects liver cells against free radical-induced oxidative stress and apoptosis *in vitro* and *in vivo* through Nrf2-dependent mechanism

M. Gokila Vani[1], K.J. Senthil Kumar[2], Jiunn-Wang Liao[3], Shih-Chang Chien[4], Jeng-Leum Mau[5], Shen-Shih Chiang[5], Chin-Chung Lin[6], Yueh-Hsiung Kuo[7,8], Sheng-Yang Wang[1,9]*

[1]*Department of Forestry, National Chung Hsing University, Taichung, Taiwan.*

[2]*Department of Cosmeceutics, China Medical University, Taichung, Taiwan.*

[3]*Graduate Institute of Veterinary Pathology, National Chung Hsing University, Taichung, Taiwan.*

[4]*The Experimental Forest Management Office, National Chung-Hsing University*

[5]*Department of Food Science and Biotechnology, National Chung Hsing University, Taichung, Taiwan.*

[6] *Taiwan Leader Biotech Company, Taipei, Taiwan*

[7]*Graduate Institute of Chinese Pharmaceutical Science, China Medical University, Taichung, Taiwan*

[8]*Department of Biotechnology, Asia University, Taichung 413, Taiwan.*

[9]*Agricultural Biotechnology Research Center, Academia Sinica, Taipei, Taiwan.*

*Corresponding author. Tel.: +886-4-22840345-138., Fax.: +886-4-22873628

E-mail address: taiwanfir@dragon.nchu.edu.tw (S-Y Wang).

Table. S1 Antibodies used for immunoblotting

Name of antibody	Supplier
Rabbit polyclonal anti-Nrf-2	Abcam (Cambridge, UK)
Mouse monoclonal anti-HO-1	Abcam (Cambridge, UK)
Rabbit polyclonal anti-caspase-4	BioVision (Milpitas, CA)
Human monoclonal anti-HSP70	BD Biosciences (San Jose, CA)
Rabbit monoclonal Histone H3	Cell Signaling Technology (Danvers, MA)
Rabbit monoclonal anti-JNK	Cell Signaling Technology (Danvers, MA)
Rabbit monoclonal anti-p-JNK	Cell Signaling Technology (Danvers, MA)
Rabbit monoclonal anti-p38MAPK	Cell Signaling Technology (Danvers, MA)
Rabbit monoclonal anti-p-p38MAPK	Cell Signaling Technology (Danvers, MA)
Rabbit monoclonal anti-p-ERK1/2	Cell Signaling Technology (Danvers, MA)
Rabbit monoclonal anti ERK1/2	Cell Signaling Technology (Danvers, MA)
Rabbit polyclonal anti-p-PI3K	Cell Signaling Technology (Danvers, MA)
Rabbit monoclonal anti-PI3K	Cell Signaling Technology (Danvers, MA)
Mouse monoclonal anti-caspase-9	Cell Signaling Technology (Danvers, MA)
Rabbit monoclonal anti-PARP	Cell Signaling Technology (Danvers, MA)
Anti-rabbit IgG- HRP conjucated	Cell Signaling Technology (Danvers, MA)
Anti-mouse IgG- HRP conjucated	Cell Signaling Technology (Danvers, MA)
Rabbit polyclonal anti-γ-GCLC	GeneTex (Irvine, CA)
Rabbit polyclonal anti-Cu/Zn-SOD	Millipore (Billerica, MA)
Rabbit polyclonal anti-caspase-12	Millipore (Billerica, MA)
Goat polyclonal anti-NQO-1	Santa Cruz Biotechnology (Heidelberg, Germany)
Mouse monoclonal anti-β-actin	Santa Cruz Biotechnology (Heidelberg, Germany)
Mouse monoclonal anti-p-PKC	Santa Cruz Biotechnology (Heidelberg, Germany)
Rabbit polyclonal anti-cytochrome c	Santa Cruz Biotechnology (Heidelberg, Germany)
Rabbit polyclonal anti-caspase-3	Santa Cruz Biotechnology (Heidelberg, Germany)
Mouse monoclonal anti-Bax	Santa Cruz Biotechnology (Heidelberg, Germany)
Anti-goat IgG-HRP conjucated	Santa Cruz Biotechnology (Heidelberg, Germany)

Table. S2 Oligonucleotides used for RT-PCR and Q-PCR

Name	Sequence	Reference
HO-1	F: 5'- TGC GGT GCA GCT CTT CTG-3'	(Liu et a., 2004)
	R: 5'- GCA ACC CGA CAG CAT GC-3'	
NQO-1	F: 5'- CGC AGA CCT TGT GAT ATT CCA G-3'	(Abdelhamid et al., 2010)
	R: 5'- CGT TTC TTC CAT CCT TCC AGG-3'	
γ-GCLC	F: 5'- AGT TCA ATA CAG TTG AGG-3'	(Kim et al., 2006)
	R: 5'- TAC TGA TCC TAT AGT TAT-3-3'	
Nrf2	F: 5'- AAC CAC CCT GAA AGC ACA GC-3'	(Abdelhamid et al., 2010)
	R: 5'- TGA AAT GCC GGA GTC AGA ATC-3'	
β-actin	F: 5'- ACC CAC ACT GTG CCC ATC TA-3'	(Liu al., 2004)
	R: 5'- CGG AAC CGC TCA TTG CC-3'	

References

Liu, L.; Yan, H.; Zhang, W.; Yao, P.; Zhang, X.; Sun, X. Induction of heme oxygenase-1 inhuman hepatoma cells to protect them from ethanol-induced cytotoxicity. *Biomed. Environ. Sci***17:** 315-326; 2004.

Abdelhamid, G.; Anwar-Mohamed, A.; Elmazar, M. M.; El-Kadi, A. O. Modulation of NAD(P)H:quinone oxidoreductase by vanadium in human hepatoma HepG2 cells. *Toxicol. In Vitro***24:**1554-61; 2010.

Kim, J. Y.; Yim, J. H.; Cho, J. H.; Kim, J. H.; Ko, J. H.; Kim, S. M.; Park, S.; Park, J. H. Adrenomedullin regulates cellular glutathione content via modulation of gamma-glutamate-cysteine ligase catalytic subunit expression. Endocrinology **147:**1357-64; 2006.

Fig. 1S

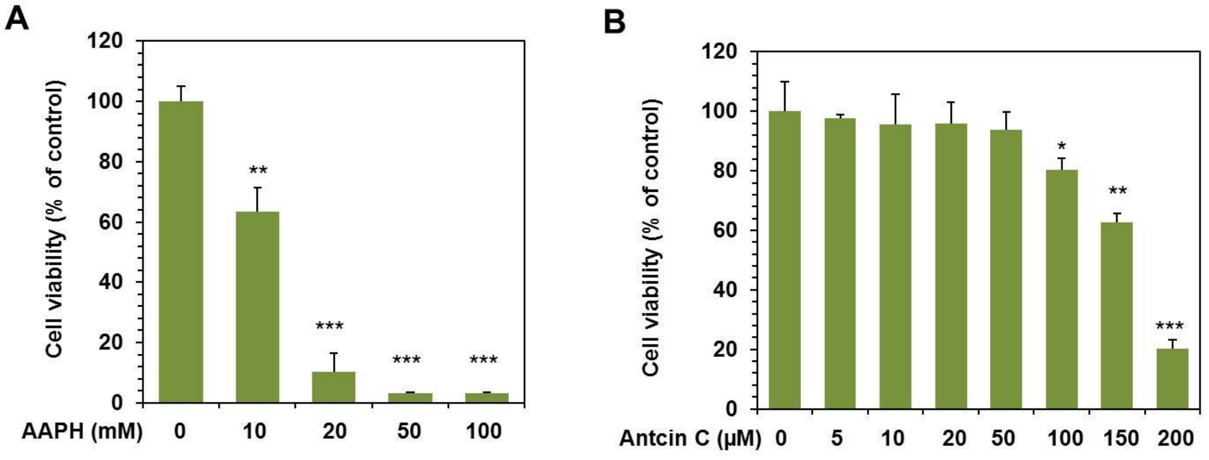

Fig. S1. Cytotoxic effect of antcin C and AAPH on cultured human hepatoma HepG2 cells. Cell viability was measured by MTT assay as described in Materials and Methods. Cells were treated with increasing concentrations of AAPH and antcin C for 24 h. (**A**) Cells treated with various concentrations of AAPH for 24 h. (**B**) Cells were treated with various concentrations of antcin C for 24 h. Percentage of viable cells was calculated with control cells. Values represent the mean ± S.D of three experiments. *$P < 0.05$, **$P < 0.01$ and ***$P < 0.001$ was considered significant for control cells.

Fig. S2.

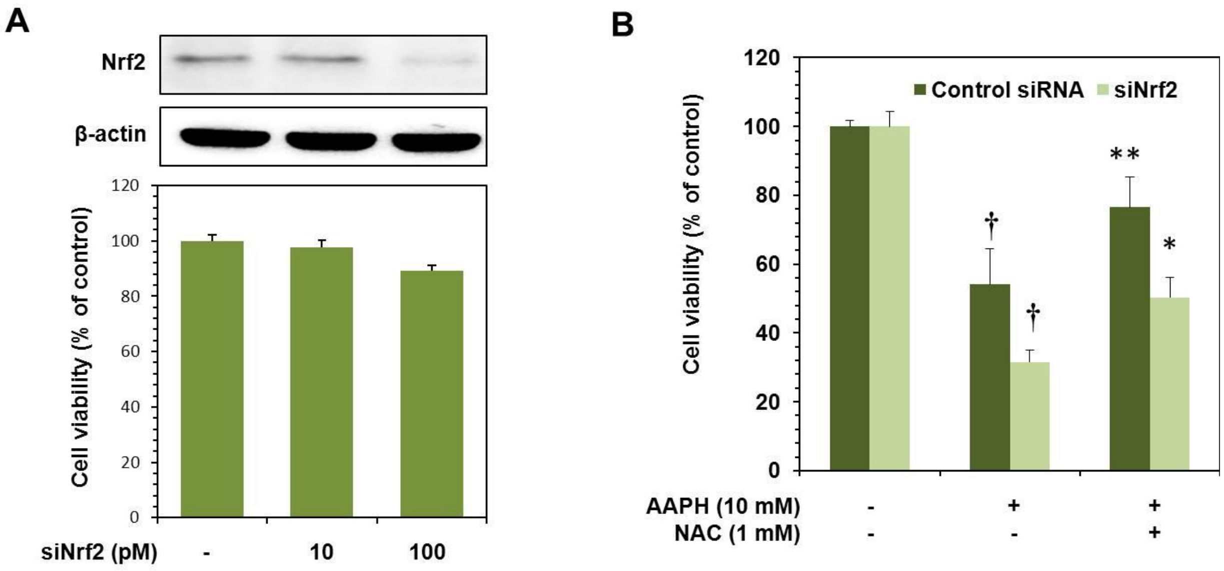

Fig. S2. SiNrf2-mediated knockdown of Nrf2 in human hepatic HepG2 cells. (A) Cells were transfected with increasing concentrations of siNrf2 (10-100 pM) for 24 h. The protein level of Nrf2 was examined by western blotting and cell survival was measured by MTT assay. (B) HepG2 cells were transfected with specific siRNA (10 pM) against Nrf2 or a non-silencing control. Following transfection for 24 h, the cells were incubated with or without NAC (1 mM) and AAPH (10 mM) for 24 h. Cell survival was examined by MTT assay. Values represent the mean ± SD of three independent experiments. *$P < 0.05$, **$P < 0.01$ was considered significant for AAPH *vs.* samples. $^{\dagger}P < 0.01$ was considered significant for control *vs.* AAPH.

Fig. S3.

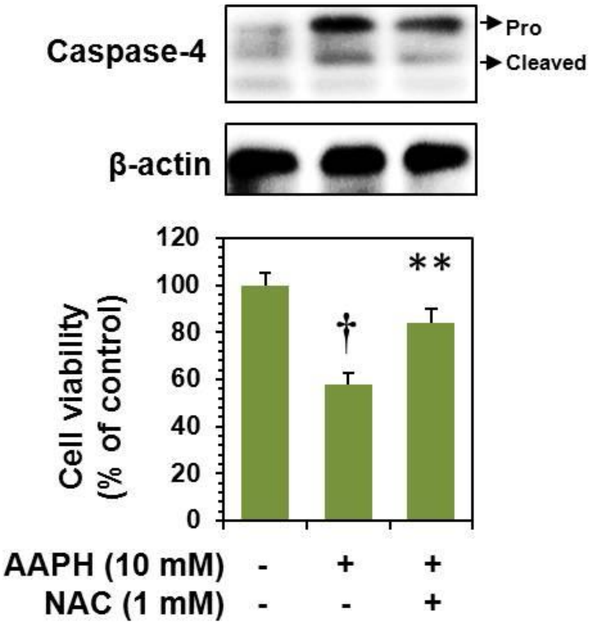

Fig. S3. NAC protects HepG2 cells from AAPH-induced apoptosis through suppression of ER stress-mediated apoptosis followed by inhibition of ROS. Cells were pretreated with NAC (1 mM) for 2 h, and then oxidative stress was induced by AAPH (10 mM) for 2-24 h. The effects of NAC on AAPH-induced ER-stress marker protein caspase 4 were examined by western blotting and cell survival was examined by MTT assay. Values represent the mean ± SD of three independent experiments. **$P < 0.01$ was considered significant for AAPH *vs.* samples. $^{\dagger}P < 0.05$ was considered significant for control *vs*. AAPH.
